# The effect of Alzheimer’s biomarker positivity on neuropsychological networks

**DOI:** 10.1093/braincomms/fcag015

**Published:** 2026-01-21

**Authors:** Laura M Wright, Matteo De Marco, Cameron E Ferguson

**Affiliations:** School of Psychology, Faculty of Medical Sciences, Newcastle University, Newcastle-Upon-Tyne NE1 7RU, UK; Department of Psychology, College of Health, Medicine and Life Sciences, Brunel University of London, Uxbridge,Middlesex UB8 3PH, UK; School of Psychological Science, University of Bristol, Bristol BS8 1TU, UK

**Keywords:** amyloid, tauopathy, graph theory, cognitive network, fluency

## Abstract

Although network neuropsychology is a promising approach to the study of clinical profiles, the link between Alzheimer’s disease (AD) biomarkers and neuropsychological networks is still undetermined. We hypothesized that network differences would exist between biomarker-positive and biomarker-negative participants, and that these would be driven by network nodes corresponding to performance on tests of episodic memory, as this is the cognitive domain most distinctively affected by AD since the earliest clinical stages. In this case–control study, we investigated sub-cohorts of individuals who had been (i) enrolled in the National Alzheimer’s Coordinating Center initiative and (ii) tested with Version 3 of the Uniform Data Set neuropsychological battery (i.e. consisting of 11 tests). These included 1263 ‘β-amyloid positive’ (A+), 1594 ‘β-amyloid negative’ (A-), 442 ‘β-amyloid and hyperphosphorylated tau positive’ (A + T+) and 734 ‘β-amyloid and hyperphosphorylated tau negative’ (A-T-) participants. We first calculated neuropsychological residuals by regressing out age, years of education, sex, Clinical Dementia Rating scores and timepoint distance between neuropsychological and biomarker assessment. Secondly, we used rank-based correlations to define conditional associations across all pairs of test scores (i.e. the nodes of the network). Thirdly, we imposed a penalty (i.e. via the Least Absolute Shrinkage and Selection Operator method) to control for network sparsity. We then tested for differences in global network metrics and node centrality between A+ and A− and between A+T+ and A−T− participants using permutation-based inferential models. Differences were found between biomarker-positive and biomarker-negative sub-cohorts in global network metrics but, contrarily to our hypothesis, no differences were found in relation to episodic memory nodes. A significant node difference, however, was instead found in relation to category fluency (i.e. a test of semantic memory), with increased centrality observed among A+ participants. A similar, yet non-significant trend was also observed between A+T+ and A−T− participants. Network neuropsychology can complement and expand the study of cognitive performance carried out via ‘traditional’ univariate approaches. While univariate analyses reveal episodic memory decline in people with AD, this is not accompanied by any abnormalities at a neuropsychological network level. Our findings, however, highlight the importance of semantic memory alterations in A+ individuals. The wide set of neural and cognitive resources that sustain semantic memory may play a supportive role in the presence of neuropathology.

## Teaser

Wright *et al*. show that people with brain amyloidosis have significant alterations in their neuropsychological network, when this is analysed with the methods of graph theory. Moreover, their performance in ‘Semantic Memory’ (that is, memory for knowledge) had significantly higher levels of network connectivity (or ‘centrality’).

## Graphical Abstract

**Graphical Abstract:**
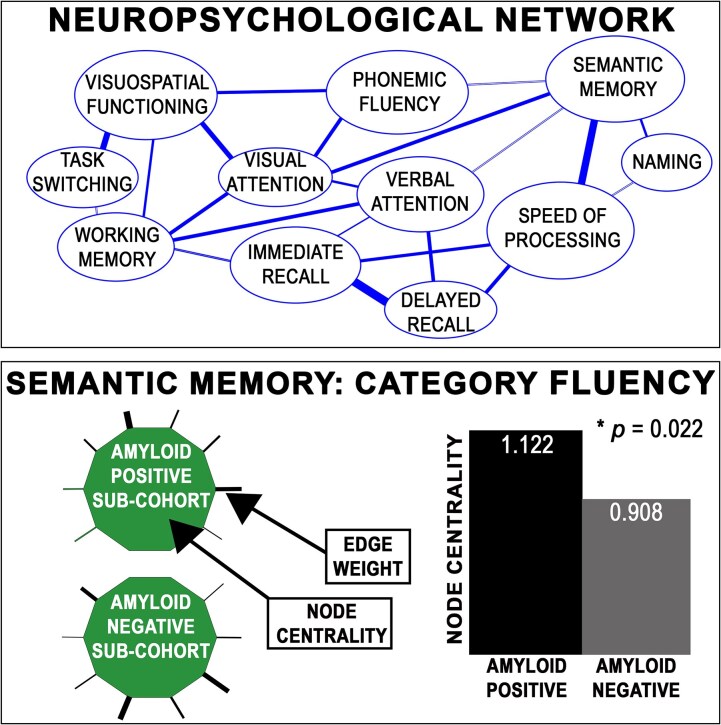

## Introduction

As the leading cause of dementia worldwide,^1^ the pursuit for earlier identification of Alzheimer’s disease (AD) remains at the forefront of research. Diagnostic protocols have recently shifted from classic clinical approaches towards biomarker definition. The ATN framework,^2,3^ where ‘A’ = Amyloid β (Aβ), ‘T’ = tau and ‘N’ = neurodegeneration, outlines an unbiased biological construct for classifying AD based on pathological markers at symptomatic and pre-symptomatic disease stages. More recently, plasma biomarkers have shown similar promise for identifying AD pathology *in vivo.*^4^ However, the application of biological frameworks in clinical settings is subject to significant debate, owing to uncertainty surrounding the prognosis of biomarker-positive cognitively unimpaired individuals who may never develop the clinical syndrome.^5,6^ While the criteria of the National Institute of Aging-Alzheimer’s Association define Aβ-positive asymptomatic individuals as having preclinical AD,^2,3^ clinical recommendations from the International Working Group consider biomarker positivity in asymptomatic individuals an indication of AD risk rather than diagnosis.^7-9^ Accumulation of AD biomarkers decades prior to observable changes in cognition emphasises the enduring need for sensitive phenotypic correlates of biomarker positivity to corroborate diagnosis in the earliest stages.^6^

In clinical stages, the relationship between AD pathology burden and cognitive function is most heavily mediated by neurofibrillary tangles (NFTs), an aggregate of hyperphosphorylated tau.^8-12^ Although studies often fail to demonstrate a linear relationship between Aβ burden and cognitive impairment, Aβ positivity is a significant predictor of later cognitive impairment and disease progression in prodromal and preclinical populations,^13,14^ emphasizing its utility in identifying AD-related pathologic change and dementia risk. Cognitive measures have also demonstrated an ability to predict progression to Aβ positivity in individuals with sub-threshold evidence of pathology,^15,16^ evidencing the bidirectional benefits of neuropsychological markers over biomarkers alone.

Evidence shows that biomarker-positive individuals often exceed thresholds of cognitive ‘normality’, owing to a range of physiological, psychological and strategic compensatory mechanisms.^17-21^ Significant correlations have been found between early NFT deposition and cognition in otherwise unimpaired older adults,^22,23^ despite early histopathological studies suggesting these stages are asymptomatic,^24,25^ demonstrating the propensity for proteinopathies to influence cognitive function at a sub-clinical level. Cognitive reserve has been proposed as a potential mechanism involved in this process; inter-individual variance in the capacity, efficiency or flexibility of neural functions, that can support normal cognition despite age or disease-related neuronal insult.^26^ Aside from pre-morbid factors such as education,^17^ this may manifest through greater flexibility in the use of cognitive strategies,^27^ or neural compensation, particularly within networks underlying executive control.^19,28,29^ In preclinical AD, normal cognitive performance may therefore reflect reorganization in both cognitive and neural processes. Nuanced cognitive change may, therefore, be best identified not by domain-specific tasks but at the level of the cognitive network.

Cognition is far from a purely segregated set of processes. Rather, successful cognitive functioning requires a dynamic interplay between cognitive domains. Characterization of cognitive profiles according to network analysis has given rise to the development of a sub-discipline known as ‘network neuropsychology’.^30^ This has revealed observable and measurable differences in cognitive network topology at clinical, prodromal and even preclinical AD stages.^31-37^ The benefit of network models lies in their ability to detect highly nuanced changes beyond those of the test scores that typically contribute towards clinical diagnosis. Taking a non-reductionist approach allows for the identification of AD-related changes in network-level phenomena which, at a preclinical stage, may be facilitating normal performance. It is on these premises that multivariate network-based approaches can expand the study of neuropsychological functioning that is normally carried out with more standard univariate analyses.

The aim of this study was to identify differences in cognitive networks related to Aβ and tau positivity. We hypothesized that differences in network topology exist between individuals testing positive and negative for AD biomarkers relating to their increased risk of dementia. Based on extensive evidence showing episodic memory to be the domain most heavily and consistently impacted by AD pathology,^23,38^ we expected the largest differences in network metrics to be identified in tasks evaluating this function.

## Material and methods

A case–cohort study was designed to test the study hypothesis. Methods are reported in line with guidelines on psychological network analyses.^39^

### Cohort selection

The National Alzheimer’s Coordinating Center (NACC) initiative (https://naccdata.org/) is a freely available repository of data coordinated and curated by the National Institute of Aging, via the NIA Alzheimer's Disease Research Centers (ADRC) programme. Established in 1999, NACC integrates and harmonizes clinical data from over 42 current or former ADRC across the USA.

**Figure 1:**
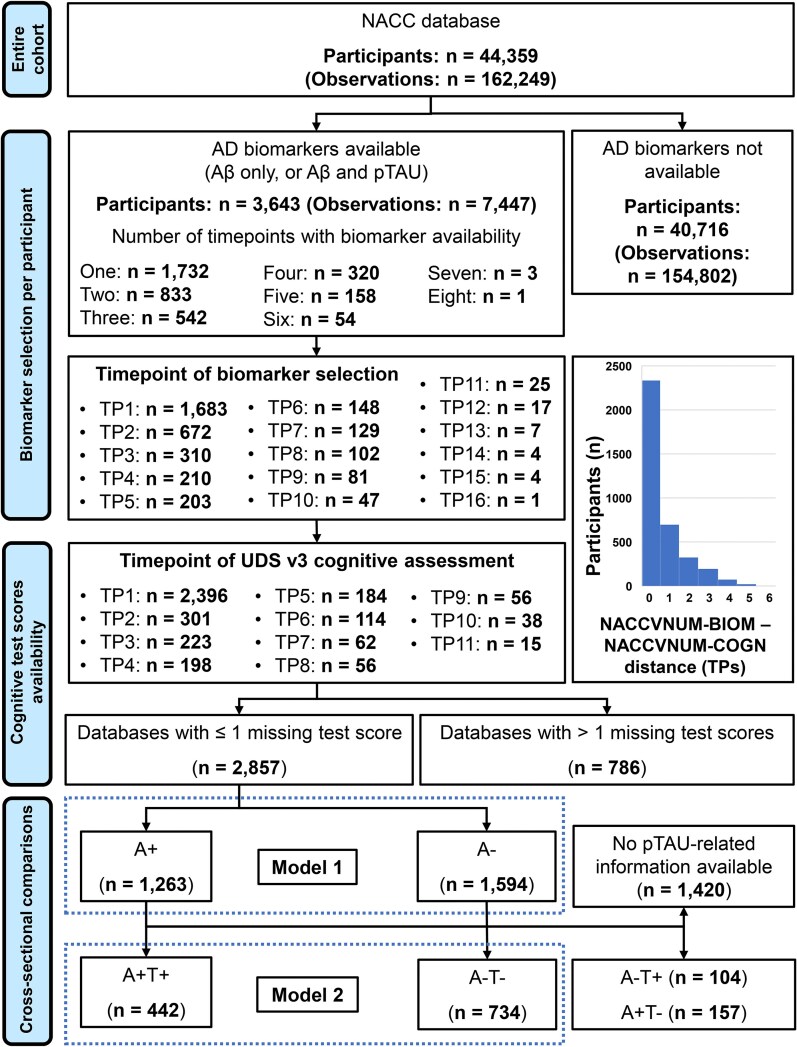
**Flowchart illustrating the process of cohort selection.** The NACC database was initially scrutinized as a function of biomarker availability. Availability of neuropsychological test scores and biomarker information was then cross-tabulated as a function of timepoint (TP), in order to define TP distances between the two clinical measures. The most commonly used version of the Uniform Data Set (UDS) battery of test was then selected for consistency across the cohort. Finally, additional criteria were applied to discard datasets with excessive missing data and discordant biomarkers. Temporal distance between TP1 and subsequent timepoints (i.e. TP2 to TP7) was calculated over the entire NACC cohort, and these averaged to 15, 29, 42, 56, 69, and 82 months. A+: Amyloid positive; A−: Amyloid negative; AD: Alzheimer’s disease. ‘NACCVNUM’ indicates the visit number at which biomarker (i.e. ‘NACCVNUM-BIOM’) and cognitive (i.e. ‘NACCVNUM-COGN’) profiles were assessed, as indexed by the NACC research data dictionary.

To address the study question, we searched the entire NACC database at one of its most recent data freezes (consisting of 44 359 unique participants and, collectively, 162 249 study visits) for study visits that included information on AD biomarkers. The main milestones of the entire process of selection are shown in Fig. 1. We initially focussed on Aβ only, relying on the ‘*AMYLPET’* and ‘*AMYLCSF*’ variables; these indicate ‘*abnormally elevated amyloid on PET*’ and ‘*abnormally low amyloid in CSF*’, respectively (i.e. definitions taken from the NACC researchers data dictionary, available at https://files.alz.washington.edu/documentation/uds3-rdd.pdf). This resulted in 7447 study visits with information on Aβ (obtained from 3643 unique participants) being retained. We defined as ‘Aβ positive’ (A+) all study visits with at least one (i.e. CSF or PET) Aβ abnormality. This led to 3662 (49.17%) visits marked as Aβ+ and 3785 (50.83%) visits marked as Aβ negative (A−). The total number of study visits attended by these 3643 participants was equal to *n* = 15 093.

As information on Aβ status for these participants was available for 1-to-8 study visits, we selected the first one to define group membership (i.e. A + or A−). We then classified the 7447 study visits to quantify those with information on phosphorylated tau status, too. To do so, we focused on the ‘*TAUPETAD’* and ‘*CSFTAU’* variables, with the same approach as that adopted for ‘*AMYLPET’* and ‘*AMYLCSF*’. A total of 3491 study visits out of 7447 (46.88%) had information on tau, with 1542 being tau positive (T+) and 1949 being tau negative (T−). The study visits with information on both biomarkers were thus distributed as follows: A−T−: *n* = 1567; A−T+: *n* = 259; A + T−: *n* = 382; A + T+: *n* = 1283.

We then reviewed all group memberships by identifying those with information on Aβ status only that also had a subsequent study visit with information on both Aβ and tau status and replaced biomarker information accordingly (i.e. 95 cases in total).

All activities carried out as part of the NACC initiative comply with the Declaration of Helsinki on ethical principles regarding human experimentation. Ethical approval was obtained from a dedicated institutional review board at each ADRC, and written informed consent was collected from each recruited participant (https://naccdata.org/requesting-data/nacc-data). Local ethical approval for secondary data analyses was received by the College of Health, Medicine and Health Sciences Ethics Committee at Brunel University of London (Review Reference: 50702-NER-Mar/2025-53965-1).

Three different batteries of neuropsychological tests have been used over the years to characterize cognitive performance of NACC participants. As per the identification of biomarker status, we identified the first available set of neuropsychological test scores for each participant. This corresponded to the first study visit for all participants. Of these assessments, 188 had been carried out using Version 1, 1059 using Version 2 and 2396 using Version 3 of the Uniform Data Set neuropsychological battery.^40^ As most cognitive assessments had been carried out using Version 3, we reviewed the database to identify the first Version-3 assessment for those originally tested with Version 1 or 2 at study visit 1.

The neuropsychological battery included 11 test scores. These are listed and briefly described in Table 1 (i.e. this highlights the three tests of episodic memory targeted by the study hypothesis). All cognitive profiles were reviewed to count the number of missing data points. Consistent with our previous work,^31^ we removed all participants who had more than one missing score. This was to ascertain that each participant would contribute to the calculation of network descriptors and metrics in a balanced way. A total of 2857 participants were retained (A−: *n* = 1594; A+: *n* = 1263). The resulting analyses used data from 30 ADRCs. Of those with information on tau, their status distributed as follows: A−T−: *n* = 734; A−T+: *n* = 104; A + T−: *n* = 157; A + T+: *n* = 442). As network modelling requires adequately large samples, the two groups with discordant biomarker status were not further considered. While the A + T− configuration defines the ‘initial’ biological stage of disease (or ‘Stage A’),^3^ an A−T + status reflects a ‘non-AD pathologic change’^2^ and was discarded for this reason. As the sub-cohort of A + T− participants was not sufficiently large for the planned analyses, we focussed on the biological presence versus absence of disease and not on any intermediate disease stage. The demographic and clinical characteristics of the final set of cohorts are described in Table 2.

**Table 1:** List and description of the neuropsychological variables included in this study

Variable	Acronym	NACC descriptor	Associated cognitive ability
Category Fluency Task	CFT	‘Total number of category-related words named in 60 s’	Semantic verbal fluency
Digit Span Forward	DSF	‘Number Span Test: Forward—Number of correct trials’	Verbal attention
Digit Span Backward	DSB	“Number Span Test: Backward—Number of correct trials’	Verbal attention/working memory
Trail Making Test Part A	TMT-A	‘Trail Making Test Part A—Total number of seconds to complete’	Visual attention
Trail Making Test Part B	TMT-B	‘Trail Making Test Part B—Total number of seconds to complete’	Visual attention/task switching
Craft Story 21 Immediate Recall	Story IR^a^	‘Craft Story 21 Recall (Immediate)—Total story units recalled, verbatim scoring’	Episodic memory
Craft Story 21 Delayed Recall	Story DR^a^	‘Craft Story 21 Recall (Delayed)—Total story units recalled, verbatim scoring’	Episodic memory
Multilingual Naming Test	MINT	‘Multilingual Naming Test—Total score’	Semantic memory/knowledge
Benson Figure Copy	Fig. Copy	‘Total score for copy of Benson figure’	Visuospatial functioning
Benson Figure Delayed Recall	Fig. DR^a^	‘Total score for 10− to 15-minute delayed drawing of Benson figure’	Episodic memory
Letter Fluency Task	LFT	‘Total number of correct F-words and L-words’	Phonemic fluency

^a^Identifies the measures of episodic memory against which the study hypothesis was tested.

**Table 2:** Description of demographic and clinical variables of the selected cohort^a^

Variable	A+	A−	Missing data	*P*	*η^2^_p_*	A+T+	A−T−	Missing data	*P*	*η^2^_p_*
Age (years)	70.32 (8.73)	69.18 (8.86)		***		67.73 (9.41)	68.14 (9.60)		0.471	
Education (years)	16.31 (2.79)	16.32 (2.78)		0.390		17.52 (9.94)	16.98 (7.96)		0.300	
Sex (F/M)	616/647	835/759		0.055		202/240	375/359		0.189	
Handedness (L/R/A/M)	132/1098/21/0	158/1398/26/0		0.882		50/376/9/7	81/635/11/7		0.614	
CDR-Global (0/0.5/1/2/3)	257/697/270/36/3	887/580/101/24/2		***		59/263/104/14/2	403/269/49/12/1		***	
MoCA	20.17 (5.95)	24.31 (4.52)	17/6	***		19.21 (6.02)	24.27 (4.60)	9/4	***	
CFT	12.67 (5.80)	16.07 (5.85)	0/0	***	0.077	11.82 (5.69)	16.03 (6.10)	0/0	***	0.101
DSF	7.15 (2.44)	7.84 (2.50)	0/0	***	0.019	6.81 (2.49)	7.79 (2.47)	0/0	***	0.039
DSB	5.44 (2.43)	6.45 (2.37)	0/2	***	0.042	5.09 (2.35)	6.38 (2.44)	0/1	***	0.063
TMT-A	52.99 (36.42)	37.39 (22.68)	0/2	***	0.064	58.42 (40.48)	36.94 (23.83)	0/2	***	0.100
TMT-B	143.64 (87.60)	98.64 (64.01)	177/73	***	0.080	152.15 (89.03)	97.88 (66.57)	81/47	***	0.106
Story IR	12.78 (8.23)	19.14 (8.20)	0/0	***	0.129	11.25 (7.57)	18.55 (8.29)	0/0	***	0.163
Story DR	8.70 (8.50)	15.95 (8.46)	0/0	***	0.153	7.49 (7.60)	15.65 (8.43)	0/0	***	0.192
MINT	27.09 (5.43)	28.75 (4.64)	5/16	***	0.026	26.54 (5.85)	28.89 (4.48)	3/12	***	0.049
Fig. Copy	13.99 (3.76)	15.33 (1.78)	0/0	***	0.053	13.36 (4.23)	15.30 (1.89)	0/0	***	0.090
Fig. DR	5.89 (4.87)	10.08 (4.09)	7/9	***	0.179	5.21 (4.71)	10.25 (4.09)	2/6	***	0.241
LFT	23.48 (9.62)	25.39 (9.72)	7/9	***	0.010	22.34 (10.00)	25.63 (10.12)	4/5	***	0.025

^a^ Means and standard deviations are shown. Between-group differences were tested with between-sample ANOVAs. A, ambidextrous; L, left; M, missing; R, right; *η²*_P_, partial eta squared.

^***^ *P* < 0.001.

Figure 1 also includes information on the exact NACC timepoints at which biomarker status information and Uniform Data Set -Version 3 cognitive assessment were selected. In the majority of cases (81.6%), the two were extracted from the same timepoint or at a distance of 1 timepoint only, while 9.5, 5.6, 2.5, 0.6 and 0.1% were extracted at a distance of 2, 3, 4, 5 or 6 timepoints, respectively.

### Neuropsychological profiles

As performance in neuropsychological tests is influenced by demographic characteristics and by clinical status, linear regression models were carried out to calculate neuropsychological residuals. Before these calculations, *TMT-A* and *TMT-B* scores were multiplied by −1, to align with the pattern among other cognitive measures where higher values indicate better performance. Age, years of education, sex, the global CDR® Dementia Staging Instrument (CDR) score and the difference (expressed in timepoints) between biomarker and cognitive assessment were regressed out to this end. A + participants scored significantly worse than A− participants on 9 out of 11 residuals at *P* < 0.001. No differences were observed in relation to *LFT* (*t_2839_* = 1.653, *P* = 0.098) or *MINT* (*t_2500.434_* = 1.658, *P* = 0.097). In the sub-cohort of participants with Aβ and tau information, A + T + participants scored significantly worse than A−T− participants on 8 out of 11 residuals at a *P* < 0.001, on 1 residual at a *P* = 0.002 and on 1 further residual at a *P* = 0.008. No differences between A + T + and A−T− individuals were found on *MINT* (*t_1425_* = 1.151, *P* = 0.250). All these descriptives were obtained via two-tailed *t*-tests.

### Network creation

We followed the methodology described by Epskamp and colleagues,^41,42^ and already applied in our previous publication,^31^ to calculate four distinct neuropsychological networks, i.e. in A+, A−, A + T+ and A−T− individuals. The *R* processing environment (version 4.2.1; https://www.r-project.org) with the *bootnet* (version 1.6; https://cran.r-project.org/web/packages/bootnet/index.html) and the *qgraph* (version 1.9.8; https://cran.r-project.org/web/packages/qgraph/qgraph.pdf) libraries were used for this purpose.

As data were not normally distributed, we used Spearman’s *rho* coefficients of correlation to calculate pairwise non-conditional associations. Spearman’s *rho* was also used to calculate conditional associations, i.e. pairwise statistical associations that are partialised for all remaining test scores, in line with recommendations.^43^ As 11 tests (i.e. the *nodes* of the network) were included in the procedures, a total of 55 (*n* × (*n*– 1)/2) associations (i.e. the *edges* of the network) were calculated. It is widely established in the neuropsychological literature that test performance tends to be positively correlated across cognitive domains,^44,45^ and the calculation of non-conditional associations reported in Supplementary Table S1 confirms this trend. Conversely, conditional associations were considerably weaker, with only 57 of the 220 measures calculated across all 4 sub-cohorts being above 0.1 or below −0.1 (Supplementary Table S1; Supplementary Fig. S1): A procedure was applied at this stage to control for the degree of sparsity and discard edges that are irrelevant to the network.^41,42^ Least Absolute Shrinkage and Selection Operator (LASSO) is a method that minimizes the equation that consists of the sum of the statistical residuals plus a *λ* penalty applied to the (absolute value of the) statistical coefficient.^46^ The choice of an adequate *λ* is based on the value that minimizes the Extended Bayesian Information Criterion (EBIC). EBIC is an extended family of the Bayesian Information Criterion (BIC) that adds to its formula a component accounting for the size of the collection of models. This additional component is regulated by the hyperparameter *γ*, which can range between 0 and 1.^47^ When *γ* is 0, the additional component is equal to 0 and EBIC = BIC, while a value of 0.5 (the value we selected) typically prioritizes specificity and is used as the default value.^43^

The application of the LASSO resulted in ∼32% of all edges being discarded (see Supplementary Table S1 for the entire set of unconditional associations, conditional associations and edge weights). To assess variability of network edges, a simulation study was carried out within each sub-cohort, by bootstrapping edge weight via *n* = 1000 random-sampling repetitions.^41^ The results indicate an excellent overlap between the model value and the bootstrapped mean and only modest variability (Supplementary Fig. S2).

### Calculation of network centrality

Network centrality can be thought of as the amount of connectivity a node shares within the network system. While a wide number of path-based centrality metrics such as *Degree* or *Betweenness Centrality* are commonly applied to unweighted networks such as those typically estimated from resting-state functional MRI,^48^ centrality metrics that are based on edge weights are instead particularly suited (and easy to interpret) for characterising weighted networks.^49^ In this study, we calculated global and nodal one-step *Expected Influence* (EI) and *Strength* (ST) as measures of network centrality. When computed on a node, EI consists of the arithmetical sum of all weights of the edges that link that node to other nodes, with negative edges retaining their negative sign in this calculation.^50^ Nodal ST is conceptually similar to Nodal EI, but the arithmetical sum is calculated on the absolute value of all edge weights. Despite the very small number of negative edges, i.e. 11 out of all 220 edges calculated across all sub-cohorts (Supplementary Table S1), we decided to investigate both EI and ST to characterize their impact. Global EI is the sum of all edge weights, with positive and negative signs maintained in the network, whereas Global ST is the absolute sum of all edge weights.

Stability of centrality metrics was assessed by recalculating these in *n* = 1000 random samples subjected to a progressively increasing (i.e. 5% to 75%) case drop.^41,42^ The correlation between model centralities and centralities obtained from random sampling was very high (Supplementary Fig. S3), with very limited variability, indicating excellent stability.

Additionally, as inter-node correlations (and, in turn, centrality) can be affected by differential node variability,^51^ we inspected the correlations between node standard deviation and both ST and EI (Supplementary Table S2). All correlations were non-significant (Supplementary Table S3), ruling out any effect of this mechanism.

To explore the subdivision of neuropsychological profiles into communities, an exploratory graph analysis was run for each network using the Louvain community-defining algorithm.^52^ This method separates subsets of highly-interconnected nodes by identifying the solution (out of 1000 iterations) that maximizes network *modularity*, i.e. a value ranging between −1 and +1 that leverages between-community and within-community edge density. A confirmatory factor analysis was then run to evaluate the fit of the community structure. The output indicated poor fit of communities, with root mean square error of approximation values exceeding 0.09, i.e. A+: 0.117; A−: 0.094; A + T+: 0.116; A−T−: 0.098. As communities were not meaningful in these sub-cohorts, these results were not analysed further.

### Statistical analysis

The network comparison test (NCT) was used to statistically compare global and nodal centrality metrics across the network models for A + and A− (Model 1) and those for A + T + and A−T− (Model 2) individuals.^53^ The permutation-based NCT works by first estimating and comparing network models and accompanying graph theory metrics (i.e. global network invariance and Global and Nodal ST/EI) for two groups (e.g. A+ and A− individuals). This gives rise to a test-statistic (see Table 3 for an explanation of the test statistics reported in this study). Next, the two data sets are merged into one larger data set, and participants are randomly reassigned to two new groups, irrespective of their A + or A− (or A + T + /A−T−) status. Two new network models, with corresponding graph theory metrics, are re-estimated and compared. This permutation process is performed 1000 times, giving rise to a null distribution, which the test statistic (i.e. a difference between the two original network models) is compared against. An alpha level of 0.05 was set, and Holm–Bonferroni correction for multiple comparisons was used. This latter correction was applied to accommodate all comparisons and not just those associated with the three episodic memory nodes (i.e. *Fig. DR*, *Story IR* and *Story DR*).

**Table 3:** Summary of NCT statistics^a^

Test Statistic	Use	Formula	Explanation
*M*	NCT-based comparison of global network structure	$M_{\omega^{1}\omega^{2}}=\underset{ij}{\max}\;|\omega_{ij}^{1}-\omega_{ij}^{2}|$	An omnibus test. Largest absolute value indicating between-group differences in edge weights (i.e. *E*-statistics)
*S*	NCT-based comparison of global centrality	$S_{\omega^{1}\omega^{2}}=\left|\sum_{i=1}^{p}\;\sum_{\;j>i}\;(|\omega_{ij}^{1}|-|\omega_{ij}^{2}|)\right|$	Invariant global ST/EI: Absolute difference in global ST/EI between groups
*E*	NCT-based comparison of edge weights	$E_{\omega^{1}\omega^{2}}=|\omega^{1}-\omega^{2}|$	Between-group absolute difference in edge weights

^a^ *ω* ^1^ and *ω*^2^ indicate the same edge of two different networks (e.g. that of the A + and that of the A− groups). EI, expected influence; ST, strength.

## Results

### Model 1: effect of amyloid positivity on cognitive networks

**Figure 2:**
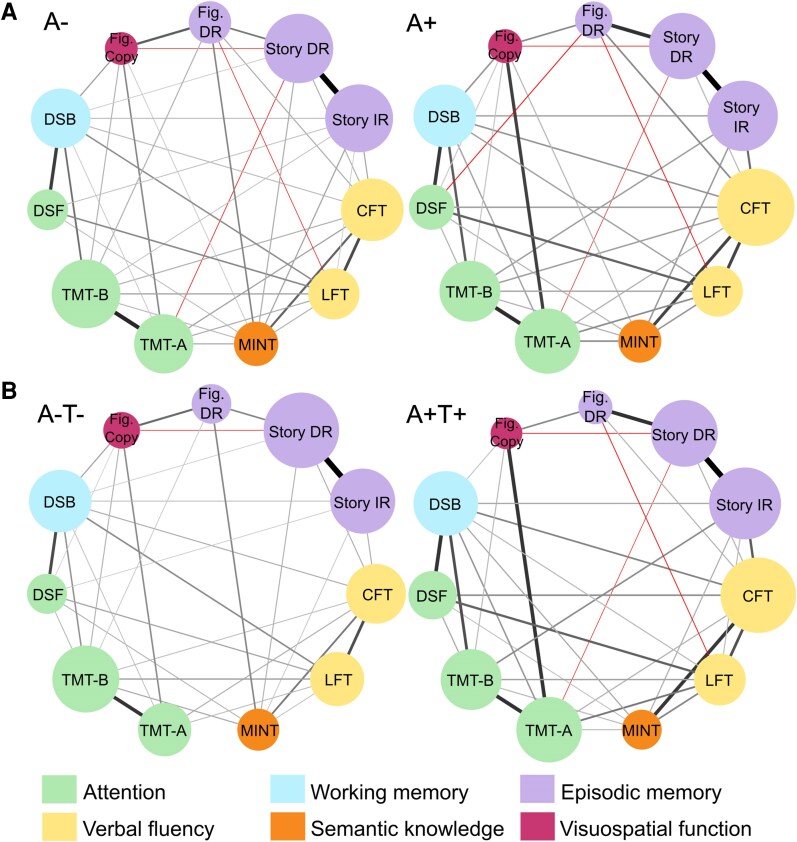
**Visual representation of the four networks estimated from the NACC database.** Negative edges are indicated in red. The thickness of the line is proportional to the weight of the edge. Nodes are represented with 11 different colours to facilitate consultation. The diameter of the node is proportional to non-standardized expected influence. All test abbreviations are defined in Table 1. (**A**) Sub-cohorts with amyloid information. A− sub-cohort: *n* = 1594; A + sub-cohort: *n* = 1263; (**B**) Sub-cohorts with amyloid and tau information. A−T− sub-cohort: *n* = 734; A + T + sub-cohort: *n* = 442. * Example outlining the difference between the two measures of centrality calculated in relation to the *Story DR* node, in the sub-cohort of A− participants (*Story DR* edge weights: CFT = 0.051; MINT = 0.057; TMT-A = −0.018; TMT-B = 0; Story IR = 0.793; DSB = 0.003; DSF = 0; Fig. Copy = −0.038; Fig. DR = 0.155; LFT = 0). $EI_{Story\;DR}=\sum edge\;weights=\;1.003;$  $ST_{Story\;DR}=\;\sum |edge\;weights|=\;1.115.$

**Figure 3:**
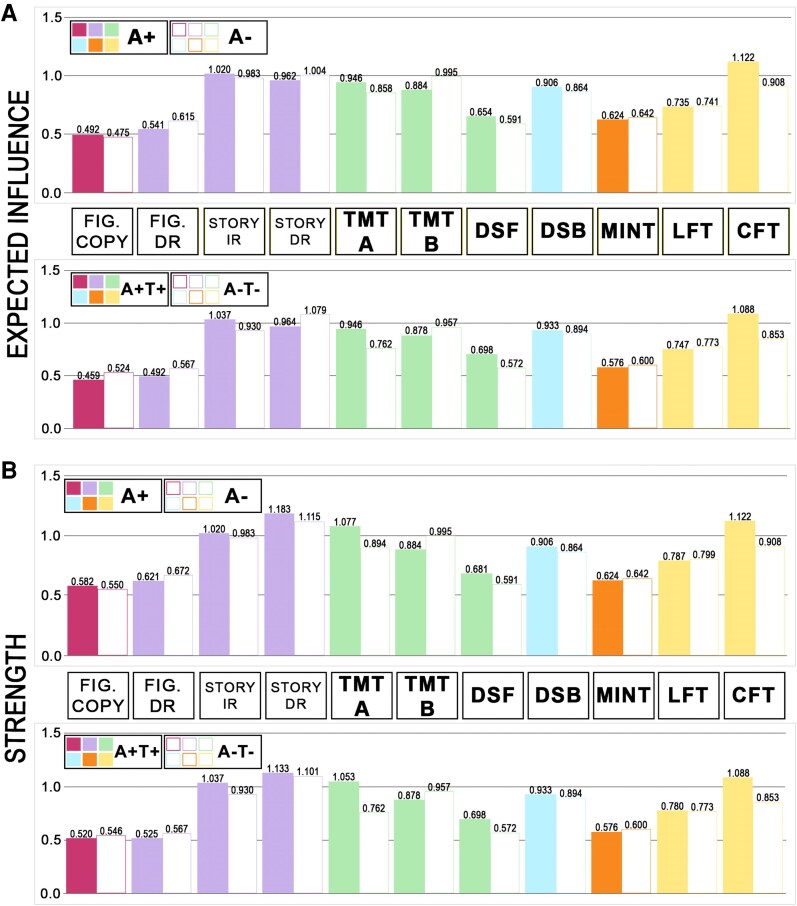
**Node centrality in each sub-cohort.** Centrality is shown separately in relation to Model 1 (i.e. A + versus A−) and Model 2 (i.e. A + T + versus A−T−). Colour coding follows the separation of test scores into distinct cognitive domains, as done in Fig. 2. Centrality measures are indicated on the y axis, i.e. EI in the upper half of the graph (**A**), ST in the lower half of the graph (**B**). The image shows non-standardized centrality metrics. For standardized centrality metrics, please see Supplementary Fig. S4. All test abbreviations are defined in Table 1. A+: amyloid positive; A−: amyloid negative; T+: tau positive; T−: tau negative. Sub-cohort information: A− sub-cohort: *n* = 1594; A + sub-cohort: *n* = 1263; A−T− sub-cohort: *n* = 734; A + T + sub-cohort: *n* = 442.

A significant difference was found between the two groups in the general structure of the network (*M* = 0.232, *P* < 0.001). Density (0.673 versus 0.709) and average edge weight (0.081 versus 0.079) were descriptively very similar across A+ and A− models. No difference was found in Global EI (*S* = 0.106, *P* = 0.140) or Global ST (*S* = 0.231, *P* = 0.200). The three episodic-memory nodes showed no differences in Nodal EI or ST between the two groups (all *P*-values > 0.05). Edge weights between *Story IR* and *Story DR* (*P* = 0.055), *Story IR* and *CFT* (*P* = 0.055) and *Story DR* and *Fig. DR* (*P* = 0.055) were marginally significantly stronger in the A + model compared to the A− model after correction for multiple comparisons. A statistically significant centrality difference was found in relation to *CFT*: A + individuals showed higher levels of both Nodal ST and Nodal EI than A− individuals (*P* = 0.022 for both centrality metrics, Holm–Bonferroni corrected). Networks are shown in Fig. 2, and non-standardized centrality for this and the other nodes are shown in Fig. 3 (while standardized centralities are shown in Supplementary Fig. S4).

**Figure 4:**
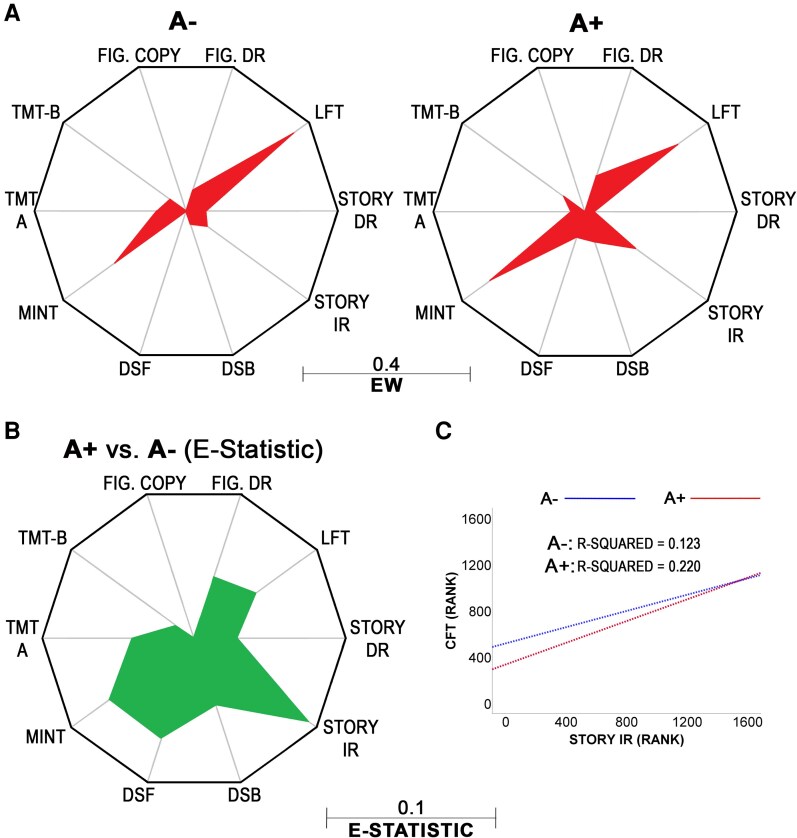
***Post hoc* characterization of *CFT* node**. (**A**) Star plots showing edge weights in relation to the only Model-1 node showing a statistically significant between-group difference. The *CFT* node is at the centre of the decagon, and the maximum weight of the circumradius is indicated below (i.e. 0.4). The figure does not show any inferential test but is simply a descriptive visualization of edge weights that are calculated prior to inferential modelling. EW: edge weights. (**B**) Star plot illustrating the size of the *E* Statistic (i.e. the absolute value of the difference in edge weight between the two groups). The largest (non-significant) difference was found in correspondence to the *Story IR*-*CFT* edge (*E* = 0.093, *P* = 0.098). The original non-conditional associations driving the differences in this edge weight are shown in (**C**). This image shows the slope of the regression lines corresponding to the rank-based correlations between CFT and IR performance in the A + and A− sub-cohorts. All test abbreviations are defined in Table 1. A+: amyloid positive; A−: amyloid negative. Sub-cohort information: A− sub-cohort: *n* = 1594; A + sub-cohort: *n* = 1263.

To characterize the nature of this difference in nodal centrality, we used the NCT to analyse the between-group differences in *CFT* edges. Although a pattern of differences was found (Fig. 4A), no edge reached statistical significance. A trend of significance was noted in correspondence to the *CFT*-*Story IR* edge (*E* = 0.093, *P* = 0.055, Fig. 4B, C).

### Model 2: effect of amyloid and tau positivity on cognitive networks

A significant difference was found between the two groups in the general structure of the network (*M* = 0.222, *P* = 0.004). Density (0.636 versus 0.618) and average edge weight (0.080 versus 0.077) were descriptively very similar across A + T + and A−T− models. No difference was found in Global EI (*S* = 0.152, *P* = 0.264) or Global ST (*S* = 0.332, *P* = 0.320). As can be seen from Fig. 3, the patterns shown by Model 2 groups were very similar to those shown by Model 1 groups. No between-group differences in EI or ST, however, were found for any of the nodes. Edge weights between *Story IR* and *Story DR* (*P* = 0.055), *Story IR* and *CFT* (*P* = 0.055) and *Story DR* and *Fig. DR* (*P* = 0.055) were marginally significantly stronger in the A+T+ model compared to the A–T– model after correction for multiple comparisons.

## Discussion

Patterns of neuropsychological network topology related to Aβ and tau positivity were tested. Retrospective examination of the NACC dataset identified 2857 participants with available biomarker and neuropsychological data. Networks created using data from 11 neuropsychological tests after controlling for demographic and clinical variables were compared between A + and A− groups, and A + T + and A−T− groups. In both cases, biomarker positivity was associated with a significant difference in general network structure. Contrary to our initial hypothesis, we found no significant differences in network metrics related to episodic memory. A significant difference in centrality was evident between A+ and A− groups for the *CFT*, with a trend indicating this was largely driven by edge weight between *CFT* and *Story IR* in the context of the wider network model. Such a finding was evident for both ST and EI, reflecting the absence of negative edges in relation to *CFT*. Although this finding only emerged as a trend approaching significance in the model including tau, the pattern of edges contributing to *CFT* centrality was similar in both models.

Network analysis literature on cognition in AD is currently in the relatively nascent stages. As such our hypothesis that episodic nodes will demonstrate the greatest between-group differences was theoretically formed on the basis of the widely reported declines in episodic memory related to AD pathology.^23,38^ In the current study, significant differences were observed between biomarker-negative and biomarker-positive groups across all cognitive domains, including every episodic memory task. What these findings therefore emphasise is the utility of network metrics in capturing characteristic differences in cognitive profiles beyond the level of numerical differences in task scores. Previous network studies have similarly identified comparable strength centralities of episodic memory nodes within cognitively normal older adults as in clinical AD groups.^32,34^ Such findings therefore suggest that episodic memory performance may be influential in the cognitive network of healthy older adults even in the absence of observable declines in task performance.

*CFTs* show moderate-to-high EI,^31^ ST,^32,34^ closeness and/or betweenness centrality,^34,35,37^ in cognitive network models of clinical AD dementia. High centrality of *CFTs* in cognitive networks may reflect how multifaceted this task is. *CFTs* are characterized by their interrogation of both semantic memory and executive functions,^54^ e.g. writing a shopping list by shifting across different categories. Semantic processing alone depends on converging multi-modal information from widespread modality-specific cortical areas.^55^ In their Hub-and-Spoke model,^55^ Lambon Ralph and colleagues indicate that semantic knowledge is represented in a distributed network of modality-specific brain regions, sending and receiving information to and from an amodal anterior temporal hub. This highlights the large-scale topology (and cytoarchitectural diversity) of the resources sustaining semantic processing. In addition, *CFTs* also require semantic control. Controlled retrieval processes rely on an interacting but largely separate network, cross-talking with working memory and executive functions, distributed within prefrontal and temporoparietal areas.^55^ That *CFTs* elicit such widespread cortical activations,^56^ indicates a pivotal role of semantic-executive processing in supporting neuropsychological functioning at a wider network level. Accordingly, common areas of neural activity have been identified during episodic, semantic and working memory tasks,^57-59^ and processing speed and executive functioning are suggested to contribute to verbal fluency performance and impairments in older adults.^60,61^ The multifaceted nature of *CFTs* may further explain why similar group differences in centrality metrics were not identified in the *MINT*, a task that specifically interrogates semantic knowledge. The *MINT* task, which, unlike the *CFT*, involves the presentation of an external visual cue, may elicit a purer semantic recognition response that is more readily dissociable from executive contributions to the controlled retrieval aspect of verbal fluency tasks.^55^ Similarly, despite the shared retrieval processes of *CFTs* and *LFTs*, the lack of a semantic component may explain why the centrality of the *LFT* showed no meaningful differences between groups. The two tests have different diagnostic properties, with CFT scores classifying controls and people with AD more accurately than LFT.^62^ In the present study, the main edge contributing to differences in *CFT* centrality between both A+ and A− and A+T+ and A−T− was between *CFT* and *Story IR*, with the edge between the *CFT* and *MINT* demonstrating the second largest difference. Despite classic representations of semantic and episodic memory as dissociable processes,^63^ they are now more typically understood as being interdependent,^64,65^ with episodic memory deficits being found to influence *CFT* in individuals with mediotemporal lobe amnesia.^66^ Centrality differences in *CFT* between biomarker groups appear then to be largely related to the overlap in episodic and semantic memory function, which *LFTs* do not tap into.

Recognizing the richness and complexity of neurological and neuropsychological functioning is of crucial importance in the context of AD research. When AD pathology affects the nervous system, it induces cognitive changes that reflect the complexity of the underlying neural substrate and how this ‘responds’ to pathology. Although Aβ and tau both demonstrate a propensity to disrupt the semantic network, influencing *CFT* performance at very early disease stages,^22,67^ the widely distributed and multifaceted networks that sustain performance on *CFTs* will allow for part of these resources to remain available. In this respect, greater *CFT* centrality may be explained by the mutual interaction it shares with other domains affected in AD, such as episodic memory. Experimental and neuroimaging research in conjunction with cognitive theory may clarify the source of the association between greater *CFT* centrality in the A + group.^30^

In the present study, the main edge contributing to differences in *CFT* centrality between both A + and A−, and A + T + and A−T− (although not statistically significant), was between *CFT* and *Story IR*, with edges between *CFT* and *MINT* and *DSF* demonstrating the second and third largest difference, respectively. Earlier research similarly reported that the edge between the *CFT* and immediate recall on a list-learning task was stronger in the network of early AD patients compared with prodromal AD and cognitively normal controls.^32,36^ Moreover, dimensionality analysis suggested that *CFT* formed a cluster with age, confrontation naming and immediate list-learning recall in early AD while forming a cluster with tests sensitive to attention, processing speed and executive functioning in the cognitively normal model.^68^ The cognitive network model of AD in Nevado *et al*. similarly displayed links amongst *CFT* and confrontation naming tests (both requiring semantic memory) and logical memory, while a control model featured links among *CFTs* and tasks sensitive to attention, processing speed and executive functioning.^32^ Consistent associations between *CFTs*, confrontation naming and list-learning or logical memory tasks among AD groups may be explained by mechanisms of compensation reliant on the mutual interaction of semantic and episodic memory. Semantic resources, if available, can be used, consciously or unconsciously, in support of encoding. Since Craik and Lockhart’s seminal paper,^69^ it has been widely accepted that semantically-mediated encoding facilitates memory performance even in anterograde memory paradigms such as immediate logical memory recall, e.g.^70^ Given A + individuals are subjected to the effects of a pathology not present in A– individuals, they may benefit from increased crosstalk between verbal encoding and semantic processing, paralleling the compensatory neural activity that has been identified in A + groups.^18^ Greater compensatory activation of the semantic network, identified in clinical AD stages,^71^ may similarly explain differences in edge weight between semantic naming and *CFT* tasks. Ferguson^32,67^ hypothesized that semantic networks underlying *CFT* performance support the acquisition of word-list memoranda in early AD. This could also apply to logical memory, given that the information to be remembered is semantically rich and often consists of memoranda characterized by ‘semantic relatedness’, a property of verbal material that people with AD dementia may benefit from during the learning phase.^72^ However, stronger associations between *CFT* and episodic memory variables in network models could also reflect shared mediotemporal pathological substrates of semantic and episodic memory deficits, rather than compensatory relationships *per se*.^67^ Indeed, Tosi *et al*.^35^ suggested that the high centrality of category fluency in their AD network reflected temporal-lobe semantic degradation.^54,73^

Despite A + versus A− comparisons aligning clearly with studies of clinically manifest AD groups,^32-36,68^ No significant centrality differences were seen between A + T + and A−T− sub-cohorts. At both node and edge levels, however, the pattern of findings was similar across both biomarker-positive groups. The greatest difference in centrality compared with biomarker-negative groups was in *CFT* in both A + and A + T +, and the largest edge-weight differences underlying this were seen in the same three edges between the *CFT* and *Story IR*, *MINT* and *DSF*. It is possible, therefore, that the limited findings reflect a methodological aspect rather than a true negative result. Firstly, our A + T + sample comprised a sub-group of the larger A + group, for which tau measurements were not all available. It is likely that a large proportion categorized as A + who did not have tau data were also T + . We cannot, therefore, assume that findings in the A + group do not reflect similar differences in A + T + . Secondly, the smaller size of the Model 2 sub-cohort may have impacted the strength of network comparisons. Importantly, however, this is not necessarily because of a small effect. At the level of pairwise correlations, in fact, we found highly similar coefficients produced in biomarker-positive groups, even when Model 2 sub-cohorts were reduced by as much as 75%, indicating that the basic building blocks of this methodology were replicated even with more modest sample sizes. This also suggests good generalisability of these results. Overall, the trends observed in our A + T + versus A−T− analyses require replication in larger samples.

Previous network studies have indicated that the centrality of nodes within psychopathology networks may be indicative of features that may be predictive of future decline or be influential targets for intervention.^74^ Similarly, the translational relevance of node centrality in cognitive networks lies in the potential to provide a quantitative approach to determine the relevance of specific cognitive functions within the wider network. Degradation of highly central, and therefore highly influential nodes,^74^ may be hypothesized to be of greater detriment to global cognitive function. Conversely, preservation of influential cognitive functions may serve to improve or stabilize function in other domains. The clinical relevance of node centrality in cognitive network remains to be determined. However, investigation into the predictive value of highly central cognitive domains for either dementia onset or improvements associated with cognitive stimulation provides meaningful avenues for future clinical research. The findings of the present study indicate that semantic fluency tasks may be of particular relevance in predicting or slowing progressive global cognitive decline in individuals testing positive for AD biomarkers.

The analyses also reported significant differences between biomarker-positive and biomarker-negative participants in global network invariance. Although this finding results from an omnibus test (and, therefore, cannot be pinpointed to specific nodes or edges), it can be seen as an indicator that AD pathology acts as a general disturbance to the neuropsychological network, presumably due to the effect it has on functional connectivity within and between large-scale brain networks.

Some limitations should be considered. As this is the first study to investigate the link between neuropsychological networks and biological markers of AD recognized by the latest diagnostic criteria,^2,3,7^ we exclusively adhered to core biological diagnostic criteria to categorize our sub-cohorts. It is possible that an imbalance in the distribution of participant clinical status between groups (which is to be expected, as there is often convergence between biomarker and clinical status) may have influenced the findings. To account for this, a marker of clinical severity, the global CDR score, was controlled for, and as outlined, the pattern of results across Model 1 and Model 2 was highly similar. Moreover, despite highly significant differences in cognitive function emerging from univariate comparisons between biomarkerpositive and biomarker-negative groups (with many effect sizes being medium or high, as shown in Table 2), these did not translate into vastly different cognitive networks, suggesting that changes in network metrics such as node centrality are not necessarily proportionate to cognitive deficits.^75^ While this study does not specifically address the preclinical phase of AD (as this would require additional evidence, e.g. *in vivo* evidence of tau pathology limited to the transentorhinal region,^24,25^ and/or longitudinal evidence of disease progression), we argue that network-based approaches could be useful to detect nuanced phenotypic markers of the preclinical disease stage that anticipate the onset of objective performance decline in individual tests.

A second limitation is the inability to account for the range of AD variants and mixed aetiologies that may be present within the dataset. Concurrent vascular pathology or other neurodegenerative aetiologies impacting network topology may have diluted effects specific to AD biomarker positivity.^76^ An avenue for future work will therefore be to validate these findings while accounting for possible co-morbidities.

A third limitation is represented by the temporal misalignment between neuropsychological testing (carried out at study visit 1) and biomarker measurement. While this had no effect on the biomarker-negative sub-cohorts (as those negative at a follow-up were also negative at study visit 1), it might have resulted into a small proportion of biomarker-negative individuals being misclassified as biomarker-positive, leading to the analyses being slightly more conservative.

Fourthly, although we limited the structure of our network to residualised test scores (i.e. by regressing out demographic characteristics prior to network calculation), other studies have instead included these variables as additional nodes.^32,33,35^ On this note, no methodological gold standard has yet been defined for selecting network variables. Studies that focus on neurological profiles, for instance, may define heterogeneous networks that include demographic, neuropsychological, behavioural and other clinical variables.^77^ Along these similar lines, for instance, we could have incorporated amyloid and tau status as further nodes, to test the study hypothesis via a within-network design. It is to address these methodological alternatives that road maps to the study of neuropsychological networks are currently being planned.^78^

In conclusion, the present study aimed to determine whether AD biomarker positivity is associated with observable differences in graph-theory-informed network-based cognitive profiles. This study is the first to investigate the link between AD pathophysiology and neuropsychological networks and the findings align with neurological changes occurring with the deposition of pathology. These suggest that not only are neuropsychological networks influenced by biomarkers at a general structural level but also that specific alterations in nodal centrality and edge weight reflect similar changes identified across the AD clinical spectrum. Such observations in biologically classified groups, independent of clinical severity, indicate that cognitive network topology may provide a clinically meaningful measure of change related to biomarkers among otherwise cognitively-normal groups.

## Supplementary Material

fcag015_Supplementary_Data

## Data Availability

The datasets generated and/or analysed during the current study are available at https://naccdata.org/. *R* code used for data analysis is available at https://osf.io/q3b6w/.
