# Supplementary material for: The effect of Alzheimer’s biomarker positivity on neuropsychological networks
Source: Brain Commun. 2026 Jan 21;8(1):fcag015. doi: 10.1093/braincomms/fcag015 (PMC12887736; doi:10.1093/braincomms/fcag015)
Supplement: fcag015_Supplementary_Data [file fcag015_supplementary_data.docx]

**Supplementary Figure S1**


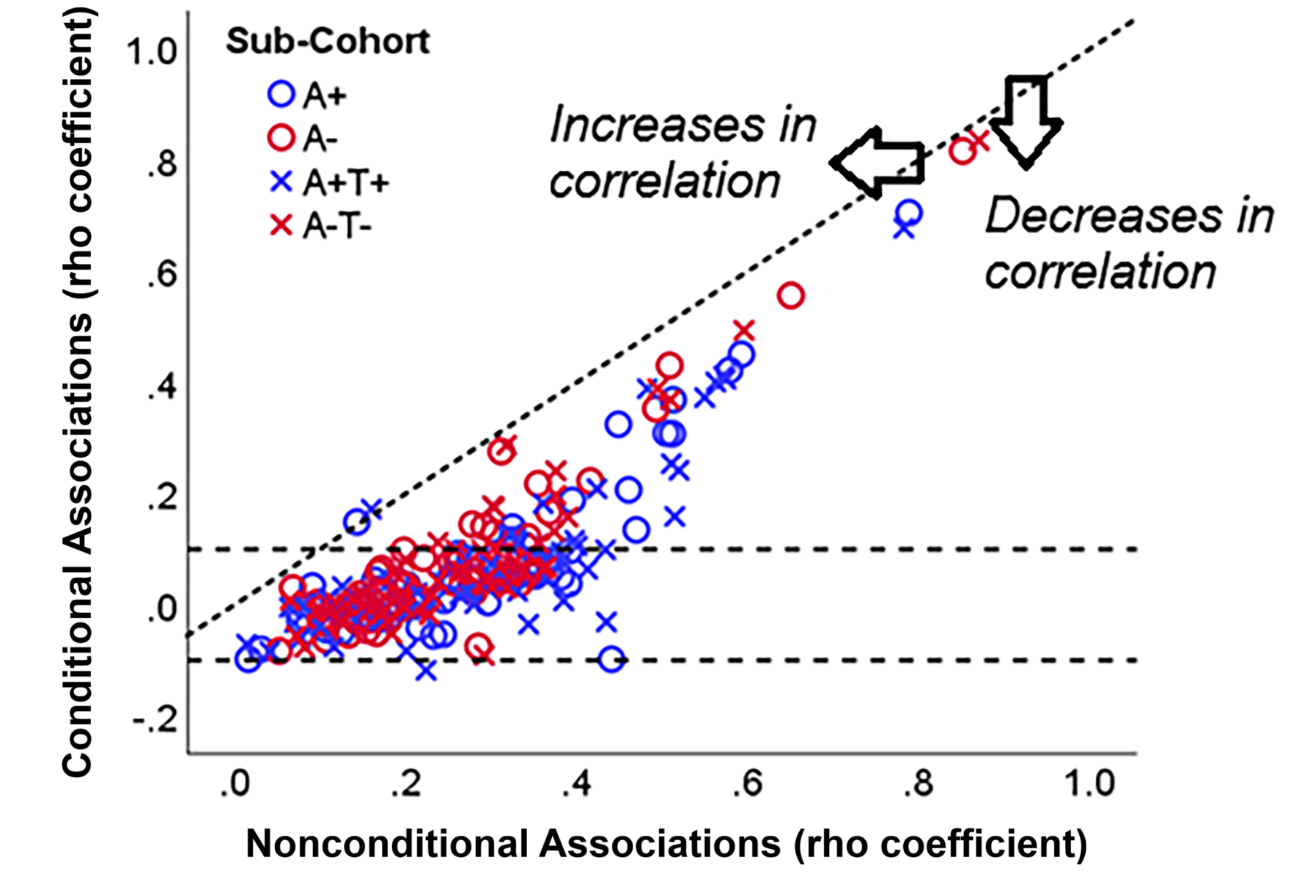


Effect of partialisation on test-to-test dependencies. The diagonal dotted line separates decreases from increases in correlational strength. The two horizontal dotted lines mark the interval of conditional associations between *rho* = -0.1 and *rho* = 0.1. Nonconditional and conditional associations are indicated in **Supplementary** **Table S1** as “NCD” and “CD”, respectively. A+: Amyloid positive; A-: Amyloid negative; T+: Tau positive; T-: Tau negative. Data points (i.e., *n* = 220) represent the 4 set of 55 edges calculated across all sub-cohorts. As this is a descriptive scatterplot, no statistical association is shown between the two variables.

**Supplementary Figure S2**

**
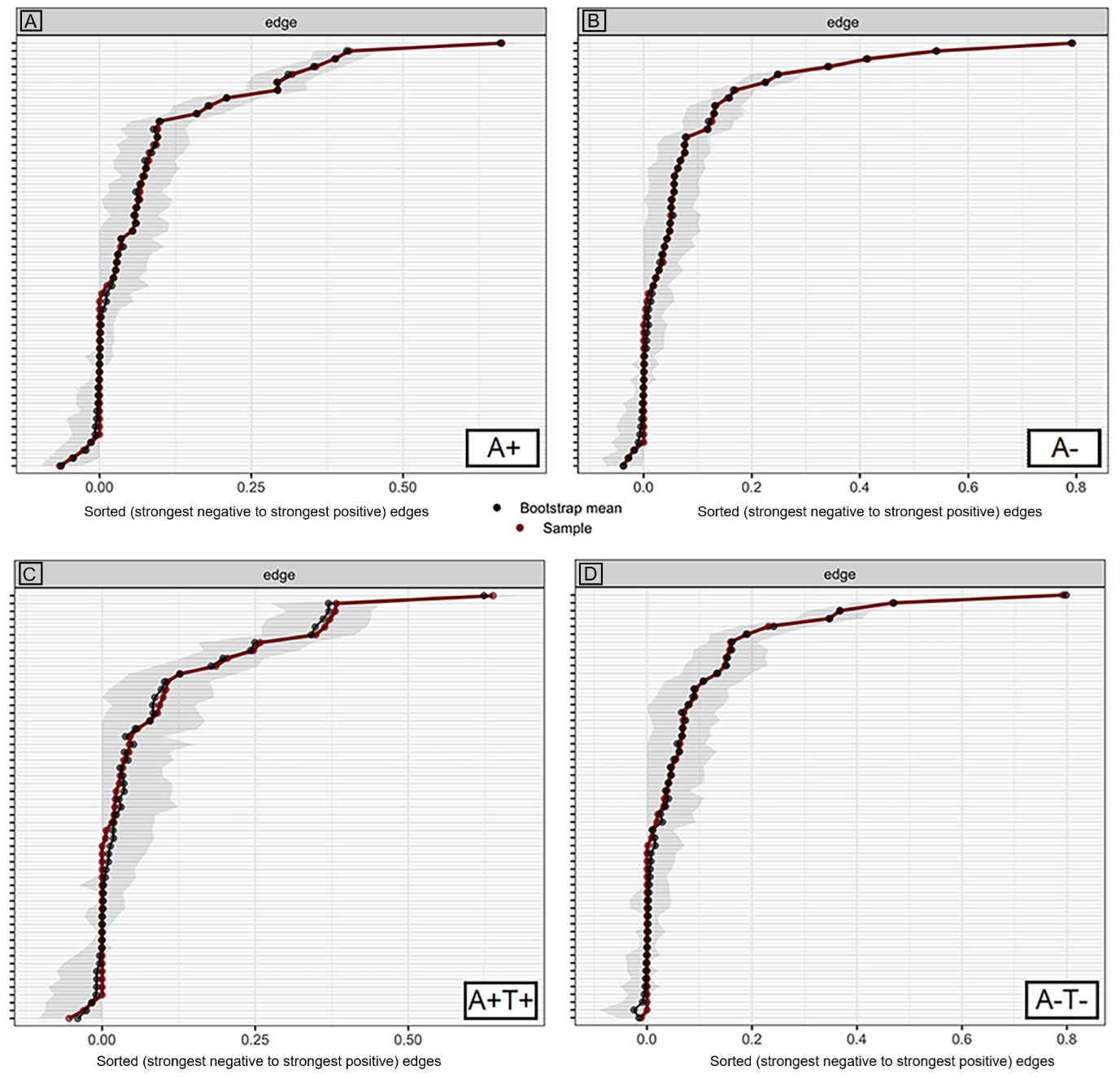
**

Simulation studies (*n* = 1,000 repetitions) showing stability of edge weights (x-axis). The 55 edges (reported on the y-axis, i.e., labels have not been included to avoid cluttering) are sorted by decreasing edge weight. The bootstrapped 95% confidence interval (shown in grey) indicates modest variability.

**Supplementary Figure S3**

**
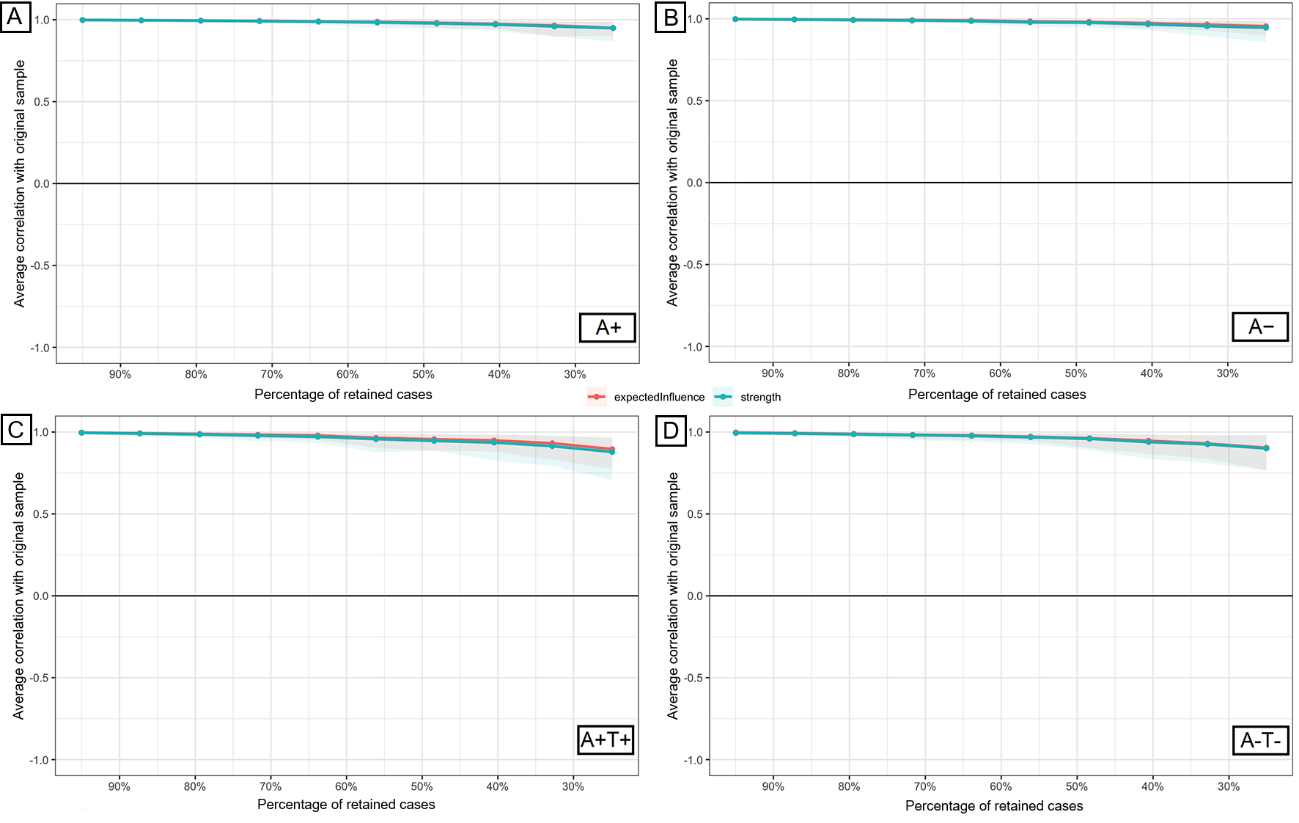
**

Simulation studies (*n* = 1,000 repetitions) testing stability of centrality metrics. A case-drop approach was used. The findings indicate robustness of EI metrics even with a drop as large as 75% of the initial sample size. A 95% confidence interval is shown around the correlational values.

**Supplementary Figure S4**


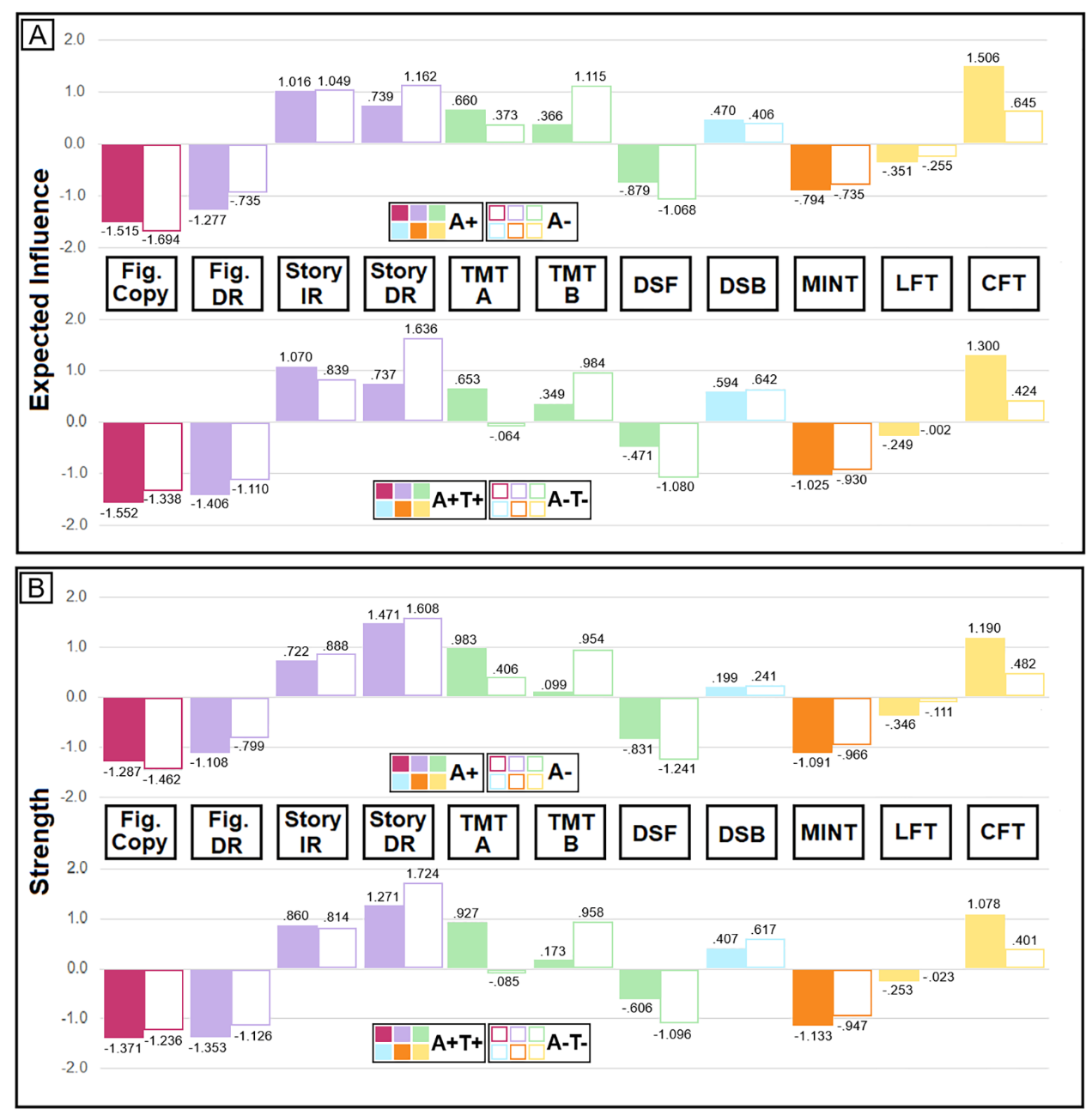


Standardised node centrality in each sub-cohort. Centrality is shown separately in relation to Model 1 (i.e., A+ vs. A-) and Model 2 (i.e., A+T+ vs. A-T-). Colour coding follows the separation of test scores into distinct cognitive domains, as done in **Figure 2**. Centrality measures are indicated on the y axis, i.e., EI in the upper half of the graph (A), ST in the lower half of the graph (B). For nonstandardised centrality metrics, please see **Figure 3**. All test abbreviations are defined in **Table 1**. A+: amyloid positive; A-: amyloid negative; T+: tau positive; T-: tau negative. Sub-cohort information: A- sub-cohort: *n* = 1,594; A+ sub-cohort: *n* = 1,263; A-T- sub-cohort: *n* = 734; A+T+ sub-cohort: *n* = 442.

**Supplementary Table S1.** Test-to-test (i.e., internodal) associations in the four sub-cohorts

| **Edge** |  | **A+** | |  |  | **A-** | |  | **A+T+-** | | |  | **A-T-** | | |
| --- | --- | --- | --- | --- | --- | --- | --- | --- | --- | --- | --- | --- | --- | --- | --- |
|  | **NCA** | **CA** | **EW** |  | **NCA** | **CA** | **EW** |  | **NCA** | **CA** | **EW** |  | **NCA** | **CA** | **EW** |
| Story DR - *CFT* | 0.391 | 0.038 | 0.023 |  | 0.344 | 0.055 | 0.051 |  | 0.413 | 0.064 | 0.030 |  | 0.365 | 0.068 | 0.067 |
| Story DR - MINT | 0.216 | **-0.041** | 0 |  | 0.323 | 0.057 | 0.057 |  | 0.201 | **-0.083** | 0 |  | 0.321 | 0.050 | 0.063 |
| Story DR - TMT-A | 0.031 | **-0.080** | **-0.066** |  | 0.107 | **-0.034** | **-0.018** |  | 0.041 | **-0.084** | **-0.054** |  | 0.096 | **-0.014** | 0 |
| Story DR - TMT-B | 0.171 | **-0.026** | 0 |  | 0.178 | **-0.008** | 0 |  | 0.204 | **-0.010** | 0 |  | 0.158 | **-0.010** | 0 |
| Story IR - *CFT* | 0.470 | 0.135 | 0.161 |  | 0.351 | 0.060 | 0.068 |  | 0.515 | 0.159 | 0.205 |  | 0.361 | 0.065 | 0.068 |
| Story IR - Story DR | 0.789 | 0.706 | 0.663 |  | 0.852 | 0.817 | 0.793 |  | 0.783 | 0.678 | 0.638 |  | 0.871 | 0.836 | 0.792 |
| Story IR - DSB | 0.284 | 0.048 | 0.059 |  | 0.212 | 0.011 | 0.036 |  | 0.331 | 0.024 | 0.046 |  | 0.238 | 0.042 | 0.038 |
| Story IR - DSF | 0.208 | 0.014 | 0 |  | 0.172 | 0.065 | 0.022 |  | 0.263 | 0.017 | 0 |  | 0.175 | 0.015 | 0.011 |
| Story IR - MINT | 0.324 | 0.117 | 0.071 |  | 0.317 | 0.044 | 0.042 |  | 0.334 | 0.112 | 0.036 |  | 0.301 | 0.031 | 0.021 |
| Story IR - TMT-A | 0.145 | 0.004 | 0 |  | 0.133 | 0.000 | 0 |  | 0.153 | **-0.011** | 0 |  | 0.099 | **-0.006** | 0 |
| Story IR - TMT-B | 0.275 | 0.082 | 0.066 |  | 0.201 | 0.034 | 0.018 |  | 0.324 | 0.128 | 0.094 |  | 0.158 | 0.007 | 0 |
| DSB - *CFT* | 0.392 | 0.097 | 0.082 |  | 0.278 | 0.040 | 0.036 |  | 0.434 | 0.099 | 0.106 |  | 0.329 | 0.078 | 0.070 |
| DSB - Story DR | 0.202 | 0.036 | 0 |  | 0.194 | 0.039 | 0.003 |  | 0.241 | 0.042 | 0 |  | 0.231 | 0.018 | 0.009 |
| DSB - MINT | 0.244 | **-0.053** | 0 |  | 0.166 | **-0.051** | 0 |  | 0.319 | 0.044 | 0.044 |  | 0.184 | **-0.052** | 0 |
| DSB - TMT-A | 0.370 | 0.080 | 0.059 |  | 0.280 | 0.023 | 0.008 |  | 0.398 | 0.116 | 0.078 |  | 0.293 | 0.044 | 0.032 |
| DSB - TMT-B | 0.461 | 0.207 | 0.210 |  | 0.368 | 0.167 | 0.170 |  | 0.520 | 0.242 | 0.258 |  | 0.374 | 0.132 | 0.135 |
| DSF - *CFT* | 0.353 | 0.069 | 0.069 |  | 0.215 | 0.000 | 0 |  | 0.401 | 0.106 | 0.090 |  | 0.228 | **-0.018** | 0 |
| DSF - Story DR | 0.133 | **-0.016** | 0 |  | 0.133 | **-0.054** | 0 |  | 0.172 | **-0.028** | 0 |  | 0.170 | 0.001 | 0 |
| DSF - DSB | 0.513 | 0.369 | 0.356 |  | 0.509 | 0.431 | 0.415 |  | 0.563 | 0.401 | 0.372 |  | 0.495 | 0.389 | 0.368 |
| DSF - MINT | 0.252 | 0.049 | 0.028 |  | 0.168 | 0.058 | 0.028 |  | 0.281 | 0.002 | 0.006 |  | 0.200 | 0.081 | 0.055 |
| DSF - TMT-A | 0.233 | **-0.056** | 0 |  | 0.158 | **-0.028** | 0 |  | 0.224 | **-0.118** | 0 |  | 0.179 | **-0.030** | 0 |
| DSF - TMT-B | 0.316 | 0.049 | 0.030 |  | 0.217 | 0.005 | 0 |  | 0.369 | 0.092 | 0.044 |  | 0.267 | 0.060 | 0.047 |
| MINT - *CFT* | 0.505 | 0.309 | 0.294 |  | 0.416 | 0.223 | 0.226 |  | 0.550 | 0.373 | 0.349 |  | 0.376 | 0.197 | 0.191 |
| MINT - TMT-A | 0.308 | 0.094 | 0.094 |  | 0.260 | 0.081 | 0.064 |  | 0.236 | 0.009 | 0.016 |  | 0.172 | **-0.014** | 0 |
| MINT - TMT-B | 0.308 | 0.041 | 0.035 |  | 0.266 | 0.034 | 0.038 |  | 0.272 | 0.024 | 0.022 |  | 0.258 | 0.099 | 0.080 |
| TMT-A - *CFT* | 0.325 | 0.051 | 0.036 |  | 0.319 | 0.087 | 0.076 |  | 0.300 | 0.073 | 0.033 |  | 0.289 | 0.098 | 0.069 |
| TMT-A - TMT-B | 0.579 | 0.422 | 0.411 |  | 0.651 | 0.557 | 0.542 |  | 0.575 | 0.406 | 0.382 |  | 0.596 | 0.494 | 0.468 |
| TMT-B - *CFT* | 0.382 | 0.050 | 0.066 |  | 0.334 | 0.039 | 0.052 |  | 0.344 | **-0.035** | 0 |  | 0.318 | 0.030 | 0.041 |
| Fig. Copy - *CFT* | 0.182 | **-0.021** | 0 |  | 0.146 | 0.007 | 0 |  | 0.138 | **-0.003** | 0 |  | 0.125 | **-0.027** | 0 |
| Fig. Copy - Story DR | 0.016 | **-0.098** | **-0.045** |  | 0.052 | **-0.083** | **-0.038** |  | 0.014 | **-0.071** | **-0.031** |  | 0.072 | **-0.056** | **-0.011** |
| Fig. Copy - Story IR | 0.091 | 0.034 | 0 |  | 0.068 | 0.030 | 0 |  | 0.064 | **-0.005** | 0 |  | 0.065 | 0.008 | 0 |
| Fig. Copy - DSB | 0.261 | 0.092 | 0.081 |  | 0.198 | 0.098 | 0.080 |  | 0.214 | 0.022 | 0.006 |  | 0.238 | 0.112 | 0.092 |
| Fig. Copy - DSF | 0.172 | 0.030 | 0.004 |  | 0.101 | **-0.008** | 0 |  | 0.126 | 0.033 | 0 |  | 0.151 | 0.018 | 0.001 |
| Fig. Copy - MINT | 0.188 | 0.045 | 0.027 |  | 0.146 | 0.019 | 0.006 |  | 0.110 | 0.012 | 0 |  | 0.131 | 0.000 | 0 |
| Fig. Copy - TMT-A | 0.449 | 0.325 | 0.317 |  | 0.278 | 0.145 | 0.129 |  | 0.483 | 0.389 | 0.364 |  | 0.304 | 0.175 | 0.149 |
| Fig. Copy - TMT-B | 0.296 | 0.004 | 0.012 |  | 0.254 | 0.041 | 0.048 |  | 0.298 | 0.027 | 0.021 |  | 0.273 | 0.055 | 0.062 |
| Fig. Copy - Fig. DR | 0.143 | 0.149 | 0.096 |  | 0.312 | 0.276 | 0.250 |  | 0.160 | 0.172 | 0.099 |  | 0.318 | 0.287 | 0.231 |
| Fig. Copy - LFT | 0.169 | **-0.022** | 0 |  | 0.100 | **-0.027** | 0 |  | 0.116 | **-0.076** | 0 |  | 0.139 | 0.009 | 0 |
| Fig. DR - *CFT* | 0.325 | 0.140 | 0.098 |  | 0.253 | 0.077 | 0.056 |  | 0.270 | 0.084 | 0.028 |  | 0.196 | 0.022 | 0 |
| Fig. DR - Story DR | 0.593 | 0.451 | 0.388 |  | 0.355 | 0.218 | 0.155 |  | 0.572 | 0.412 | 0.381 |  | 0.376 | 0.241 | 0.159 |
| Fig. DR - Story IR | 0.441 | **-0.098** | 0 |  | 0.285 | **-0.075** | 0 |  | 0.435 | **-0.031** | 0 |  | 0.292 | **-0.091** | 0 |
| Fig. DR - DSB | 0.125 | **-0.049** | 0 |  | 0.152 | 0.005 | 0 |  | 0.133 | **-0.017** | 0 |  | 0.153 | 0.001 | 0 |
| Fig. DR - DSF | 0.076 | **-0.028** | **-0.013** |  | 0.097 | 0.004 | 0 |  | 0.070 | **-0.033** | 0 |  | 0.119 | 0.003 | 0 |
| Fig. DR - MINT | 0.156 | **-0.026** | 0 |  | 0.292 | 0.141 | 0.133 |  | 0.100 | **-0.039** | 0 |  | 0.302 | 0.178 | 0.158 |
| Fig. DR - TMT-A | 0.089 | **-0.002** | 0 |  | 0.155 | **-0.046** | 0 |  | 0.080 | 0.000 | 0 |  | 0.126 | **-0.033** | 0 |
| Fig. DR - TMT-B | 0.164 | 0.044 | 0 |  | 0.221 | 0.084 | 0.050 |  | 0.135 | 0.000 | 0 |  | 0.187 | 0.057 | 0.019 |
| Fig. DR - LFT | 0.107 | **-0.045** | **-0.026** |  | 0.107 | **-0.063** | **-0.029** |  | 0.078 | **-0.060** | **-0.017** |  | 0.082 | **-0.077** | 0 |
| LFT - *CFT* | 0.512 | 0.308 | 0.294 |  | 0.493 | 0.353 | 0.343 |  | 0.511 | 0.254 | 0.247 |  | 0.510 | 0.369 | 0.347 |
| LFT - Story DR | 0.157 | **-0.018** | 0 |  | 0.194 | **-0.007** | 0 |  | 0.200 | **-0.008** | 0 |  | 0.196 | **-0.001** | 0 |
| LFT - Story IR | 0.245 | 0.008 | 0 |  | 0.215 | 0.012 | 0.003 |  | 0.311 | 0.045 | 0.019 |  | 0.200 | **-0.006** | 0 |
| LFT - DSB | 0.369 | 0.061 | 0.060 |  | 0.343 | 0.122 | 0.116 |  | 0.385 | 0.007 | 0.023 |  | 0.390 | 0.158 | 0.150 |
| LFT - DSF | 0.395 | 0.188 | 0.182 |  | 0.304 | 0.130 | 0.126 |  | 0.424 | 0.209 | 0.186 |  | 0.304 | 0.102 | 0.091 |
| LFT - MINT | 0.353 | 0.077 | 0.077 |  | 0.278 | 0.061 | 0.049 |  | 0.382 | 0.096 | 0.104 |  | 0.263 | 0.062 | 0.033 |
| LFT - TMT-A | 0.340 | 0.107 | 0.095 |  | 0.302 | 0.062 | 0.057 |  | 0.361 | 0.182 | 0.126 |  | 0.296 | 0.048 | 0.045 |
| LFT - TMT-B | 0.352 | 0.052 | 0.054 |  | 0.332 | 0.080 | 0.076 |  | 0.364 | 0.047 | 0.058 |  | 0.356 | 0.111 | 0.107 |

TMT-A and TMT-B test score residuals were multiplied by -1 prior to the calculation of correlation coefficients. NCA: Non-Conditional Associations (Standard Spearman’s *rho*-coefficient of correlation); CA: Conditional Associations (Spearman’s *rho* coefficient of partial correlation); EW: Edge Weight. Negative correlation coefficients and edge weights are indicated in bold.

**Supplementary Table S2**. Node variability and centrality metrics

|  | **A+** | | | **A-** | | | **A+T+** | | | **A-T-** | | |
| --- | --- | --- | --- | --- | --- | --- | --- | --- | --- | --- | --- | --- |
| **Node** | **SD** | **EI** | **ST** | **SD** | **EI** | **ST** | **SD** | **EI** | **ST** | **SD** | **EI** | **ST** |
| *Node standard deviations and non-standardised centrality scores* | | | | | | | | | | | | |
| *CFT* | 4.778 | 1.122 | 1.122 | 5.073 | 0.908 | 0.908 | 4.907 | 1.088 | 1.088 | 4.976 | 0.853 | 0.853 |
| DSF | 2.359 | 0.654 | 0.681 | 2.415 | 0.591 | 0.591 | 2.394 | 0.698 | 0.698 | 2.384 | 0.572 | 0.572 |
| DSB | 2.211 | 0.906 | 0.906 | 2.187 | 0.864 | 0.864 | 2.128 | 0.933 | 0.933 | 2.221 | 0.894 | 0.894 |
| TMT-A | 31.558 | 0.946 | 1.077 | 20.334 | 0.858 | 0.894 | 35.291 | 0.946 | 1.053 | 21.122 | 0.762 | 0.762 |
| TMT-B | 77.057 | 0.884 | 0.884 | 56.377 | 0.995 | 0.995 | 81.126 | 0.878 | 0.878 | 55.346 | 0.957 | 0.957 |
| Story IR | 6.914 | 1.020 | 1.020 | 7.003 | 0.983 | 0.983 | 6.730 | 1.037 | 1.037 | 6.880 | 0.930 | 0.930 |
| Story DR | 7.101 | 0.962 | 1.183 | 7.048 | 1.004 | 1.115 | 6.979 | 0.964 | 1.133 | 6.961 | 1.079 | 1.101 |
| MINT | 4.959 | 0.624 | 0.624 | 4.302 | 0.642 | 0.642 | 5.494 | 0.576 | 0.576 | 4.221 | 0.600 | 0.600 |
| Fig. Copy | 3.448 | 0.492 | 0.582 | 1.823 | 0.475 | 0.550 | 3.863 | 0.459 | 0.520 | 1.926 | 0.524 | 0.546 |
| Fig. DR | 3.990 | 0.541 | 0.621 | 3.448 | 0.615 | 0.672 | 4.079 | 0.492 | 0.525 | 3.445 | 0.567 | 0.567 |
| LFT | 8.874 | 0.735 | 0.787 | 8.915 | 0.741 | 0.799 | 9.198 | 0.747 | 0.780 | 9.187 | 0.773 | 0.773 |
| *Node standard deviations and standardised centrality scores* | | | | | | | | | | | | |
| *CFT* | 4.778 | 1.506 | 1.190 | 5.073 | 0.645 | 0.482 | 4.907 | 1.300 | 1.078 | 4.976 | 0.424 | 0.401 |
| DSF | 2.359 | -0.735 | -0.831 | 2.415 | -1.068 | -1.241 | 2.394 | -0.471 | -0.606 | 2.384 | -1.080 | -1.096 |
| DSB | 2.211 | 0.470 | 0.199 | 2.187 | 0.406 | 0.241 | 2.128 | 0.594 | 0.407 | 2.221 | 0.642 | 0.617 |
| TMT-A | 31.558 | 0.660 | 0.983 | 20.334 | 0.373 | 0.406 | 35.291 | 0.653 | 0.927 | 21.122 | -0.064 | -0.085 |
| TMT-B | 77.057 | 0.366 | 0.099 | 56.377 | 1.115 | 0.954 | 81.126 | 0.349 | 0.173 | 55.346 | 0.984 | 0.958 |
| Story IR | 6.914 | 1.016 | 0.722 | 7.003 | 1.049 | 0.888 | 6.730 | 1.070 | 0.860 | 6.880 | 0.839 | 0.814 |
| Story DR | 7.101 | 0.739 | 1.471 | 7.048 | 1.162 | 1.608 | 6.979 | 0.737 | 1.271 | 6.961 | 1.636 | 1.724 |
| MINT | 4.959 | -0.879 | -1.091 | 4.302 | -0.794 | -0.966 | 5.494 | -1.025 | -1.133 | 4.221 | -0.930 | -0.947 |
| Fig. Copy | 3.448 | -1.515 | -1.287 | 1.823 | -1.694 | -1.462 | 3.863 | -1.552 | -1.371 | 1.926 | -1.338 | -1.236 |
| Fig. DR | 3.990 | -1.277 | -1.108 | 3.448 | -0.939 | -0.799 | 4.079 | -1.406 | -1.353 | 3.445 | -1.110 | -1.126 |
| LFT | 8.874 | -0.351 | -0.346 | 8.915 | -0.255 | -0.111 | 9.198 | -0.249 | -0.253 | 9.187 | -0.002 | -0.023 |

The correlations between SD and centrality metric are reported in **Supplementary Table S3**. EI: Expected influence; SD: Standard deviation ST: Strength

**Supplementary Table S3**. Correlations between node standard deviation and centrality

|  | **A+** | | **A-** | | **A+T+** | | **A-T-** | |
| --- | --- | --- | --- | --- | --- | --- | --- | --- |
| **Sub-Cohort SD** | **EI** | **ST** | **EI** | **ST** | **EI** | **ST** | **EI** | **ST** |
| *Non-standardised centrality metrics* | | | | | | | | |
| A+ SD | 0.182 | 0.222 |  |  |  |  |  |  |
| A- SD |  |  | 0.432 | 0.472 |  |  |  |  |
| A+T+ SD |  |  |  |  | 0.195 | 0.211 |  |  |
| A-T- SD |  |  |  |  |  |  | 0.375 | 0.386 |
| *Standardised centrality metrics* | | | | | | | | |
| A+ SD | 0.182 | 0.222 |  |  |  |  |  |  |
| A- SD |  |  | 0.432 | 0.473 |  |  |  |  |
| A+T+ SD |  |  |  |  | 0.196 | 0.212 |  |  |
| A-T- SD |  |  |  |  |  |  | 0.376 | 0.387 |

Pearson’s *r* coefficients (and *p*-values) are indicated. *n* = 11 in these models.

EI: Expected influence; SD: Standard deviation ST: Strength
